# Inihibition of Glycolysis by Using a Micro/Nano-Lipid Bromopyruvic Chitosan Carrier as a Promising Tool to Improve Treatment of Hepatocellular Carcinoma

**DOI:** 10.3390/nano8010034

**Published:** 2018-01-10

**Authors:** Nemany A. Hanafy, Luciana Dini, Cinzia Citti, Giuseppe Cannazza, Stefano Leporatti

**Affiliations:** 1 CNR NANOTEC-Istituto di Nanotecnologia, 73100 Lecce, Italy; nemany.hanafy@nanotec.cnr.it (N.A.H.); cinzia.citti@gmail.com (C.C.); 2 Department of Mathematics and Physics “E. De Giorgi”, University of Salento, 73100 Lecce, Italy; 3 Department of Biological and Environmental Sciences and Technologies (DiSTeBA), University of Salento, 73100 Lecce, Italy; luciana.dini@unisalento.it (L.D.); giuseppe.cannazza@unimore.it (G.C.); 4 Life Science Department, University of Modena e Reggio Emilia, 41121 Modena, Italy

**Keywords:** nanocarrier, glycolysis, hepatocellular carcinoma (HCC), bromopyruvate

## Abstract

Glucose consumption in many types of cancer cells, in particular hepatocellular carcinoma (HCC), was followed completely by over-expression of type II hexokinase (HKII). This evidence has been used in modern pharmacotherapy to discover therapeutic target against glycolysis in cancer cells. Bromopyruvate (BrPA) exhibits antagonist property against HKII and can be used to inhibit glycolysis. However, the clinical application of BrPA is mostly combined with inhibition effect for healthy cells particularly erythrocytes. Our strategy is to encapsulate BrPA in a selected vehicle, without any leakage of BrPA out of vehicle in blood stream. This structure has been constructed from chitosan embedded into oleic acid layer and then coated by dual combination of folic acid (FA) and bovine serum albumin (BSA). With FA as specific ligand for cancer folate receptor and BSA that can be an easy binding for hepatocytes, they can raise the potential selection of carrier system.

## 1. Introduction

Increase of glucose consumption in many types of cancer cells is supported mostly by overexpression of type II hexokinase (HKII) [1]. Hence, Hexokinase (ATP: d-hexose 6-phosphotransferase) is a key enzyme that catalyzes the first step in the glycolysis pathway. This enzyme transfers a phosphate group from ATP to glucose to form glucose-6-phosphate [2]. Moreover, HKII interacts with the outer membrane protein voltage dependent anion channel (VDAC). It blocks mitochondrial inter-membrane space protein release and prevents activation of the apoptotic process [3]. This unique property has gained attention from researchers to develop new chemotherapeutic strategies targeting the glycolysis pathway in cancer cells [4]. Various inhibitors affecting the key enzymes of the glycolysis pathway have been identified. Among the glycolytic inhibitors, bromopyruvate (BrPA) shows promising anticancer activity both in vitro and in vivo. Indeed, BrPA causes regression of solid tumors by ATP depletion [5]. It has, furthermore, been shown to be effective and, indeed, curative, as a single agent against hepatic tumors in animal models [5]. The crucial problem for using BrPA in clinical application is related to its interaction with normal cells, especially erythrocytes [6]. Thus, there is an urgent need to encapsulate BrPA inside smart carriers having efficient strategies from size, shape, and targeted for cancer cells. In our previous report, BrPA attached Poly(allylamine) hydrochloride was entrapped inside CaCO_3_ rods during their fabrication. Encapsulated BrPA was absorbed by cancer cells and, furthermore, was released gradually with time as demonstrated by confocal microscopy and MTT assay [7]. However, non-specific, passive, targeting carriers can result in uptake by healthy cells. This can be minimized by the active targeting of the therapy, which has not been explored previously by this team. In our recent work, targeted hybrid lipid polymer is fabricated as an alternate assembly structure instead of liposomes. Their positive attributes (such as their tunable size, surface charge, high drug loading yield, sustained drug release profile, favorable stability in serum, good cellular targeting ability) make them a promising drug delivery vehicle for further in vivo tests. Hybrid polymeric protein carriers (HPPNCs) were assembled by using chitosan, oleic acid, and BSA-FA to produce a core-shell structure.

Chitosan is a copolymer of β-(1→4)-linked-2-acetamido-2-deoxy-d-glucopyranose and 2-amino-2-deoxy-d-glucopyranose [8]. Its unique properties, such as biodegradability, biocompatibility, nontoxicity, positively-charged, and rigid linear molecular structure make this macromolecule ideal as a drug carrier and delivery material [9]. Chitosan is soluble in aqueous solutions of various acids, but chitosan molecules have no amphiphilic property and cannot produce micelles in water. Thus, there are many reports on hydrophobic changes of chitosan, for example, palmitoyl glycol chitosan [10], deoxycholic acid-modified chitosan, [11], poly(*N*-isopropylacrylamide)-chitosan [12], linoleic acid-modified chitosan [13], linolenic acid modified chitosan [14], *N*-alkyl-*O*-sulfate chitosan [15], chitosanpolylactide graft copolymer [16], *N*-acetylchitosan, *N*-propionylchitosan, and *N*-butyrylchitosan, butanoylchitosan, hexanoy-chitosan, and benzoyl-chitosan [17]. Bovine serum albumin (BSA) is biodegradable, biocompatible, nontoxic, and not immunogenic [18], making it an ideal delivery carrier for drugs. In particular, BSA-based nanoparticles (NPs) might cause natural abundance in plasma, relative stability and inertness in biochemical pathways, availability, and a relatively benign in vivo biological fate [19]. A tumor-targeting agent, folic acid, was linked to BSA to increase the selective targeting ability of the conjugate [20]. Folic acid has been widely used as a ligand for folate receptor-mediated selective targeting and delivery of drugs into tumor cells [21]. The folate receptor has been found to be overexpressed in a wide range of tumors, and is known as a high-affinity membrane folate-binding protein, which mediates uptake of the vitamin by receptor-mediated endocytosis [22]. Recently, maximum entrapment efficiency investigation of similar systems was also performed [23] and use of SiRNA for following oral administration of chitosan/SiRNA nanoparticles was further investigated [24]. In a previous paper, we have investigated conjugation of folic acid with BSA. Whereas prior to the conjugation to BSA, FA was activated by using 1-ethyl-3-(3-dimethylaminopropyl) carbodiimide (EDAC) and NHS to trigger the binding of the carboxyl group (specifically the gamma-COOH) of FA to the free amino moieties of BSA [25]. The novelty of this work is to obtain nucleus made up of chitosan-oleic acid. Since the hydrophobic modification of chitosan that was done by coupling with fatty acid, can result in product with an amphiphilic behavior and self-aggregation. Then oleic acid grafted chitosan was inserted into a layer of albumin-FA to achieve higher drug levels in tumor tissue and to minimize side effects. 

## 2. Results and Discussion

### 2.1. Characterization

**Figure 1:**
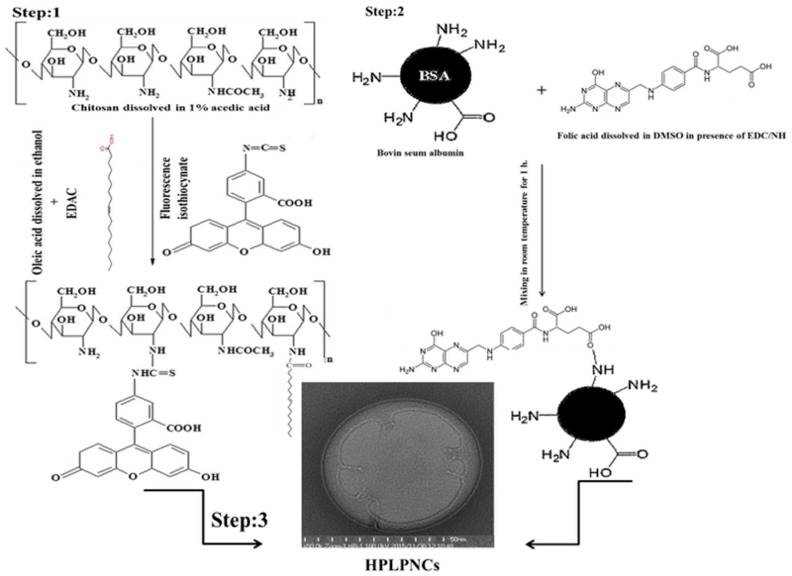
Scheme of hybrid polymer lipid protein nanocarrier structure. Step 1: self-assembly Structure of chitosan and oleic acid; step 2: conjugation folic acid with bovine serum albumin; and step 3: functionalization of chitosan grafted oleic acid surface by using BSA-FA.

Oleic acid is a mono-unsaturated fatty acid. It is able to generate reactive oxygen species (ROS) inside cells because it has free fatty acids with anti-neoplastic properties against cancer cells [26]. In this study, the active site of free fatty acid was blocked by dissolving oleic acid in alcohol [27] (e.g., in this study, ethanol was used). It was then heated for 2 h at 60 °C. Afterwards EDAC was added to activate the carboxylic group of oleic acid (see Figure 1) resulting in a homogenous esterified suspension [28]. Oleic acid (OA) was coupled to chitosan by the formation of amide linkages through the EDAC-mediated reaction with different degrees of amino substitution (DS) as described in a previous study [29] (see Figure 1, Step 1). Although chitosan molecules present no amphiphilic property and, therefore, cannot form micelles in water, chitosan chains can be modified by oleic acid by means of the introduction of carboxylic acid groups in the presence of water-soluble carbodiimide, which react with carboxyl groups of fatty acids, forming active ester intermediates. Consequently, the intermediates can react with primary amine groups of chitosan to create an amide bond. The final product of this assembly is a nano-sized self-aggregation in aqueous media [30]. These nuclei were made up of self-aggregated chitosan and oleic acid was coated by FA conjugated with BSA to target cancer cells and to minimize side effects [22].

**Figure 2:**
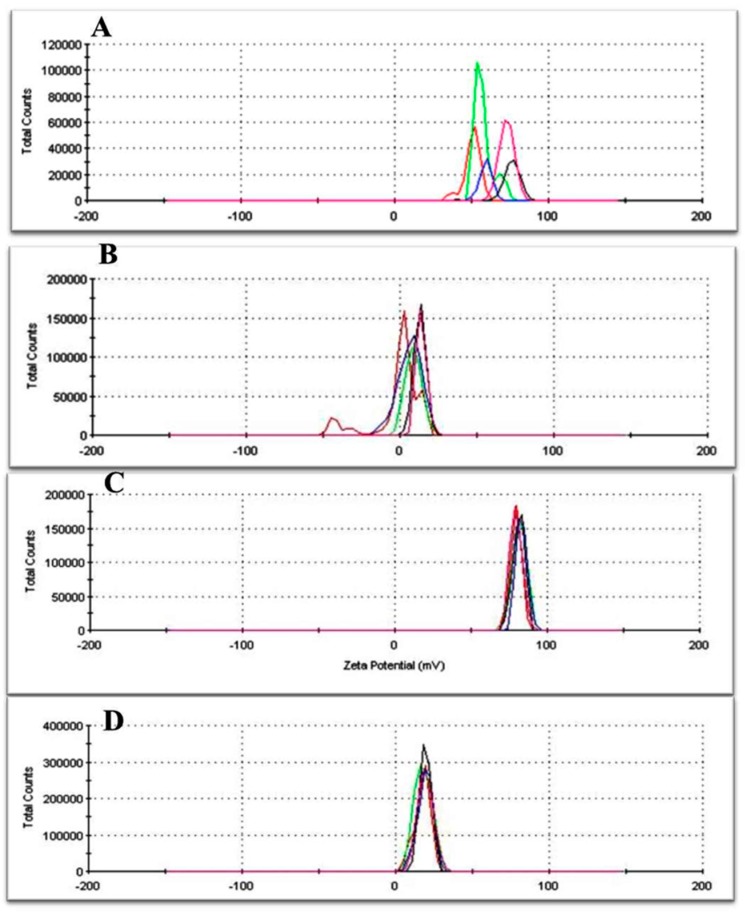
Zeta potential measurement: (**A**) chitosan solution alone; (**B**) oleic acid suspension alone; (**C**) chitosan grafted oleic acid; and (**D**) Hybrid Polymeric Lipid Protein Nanocarriers (HPLPNCs).

The zeta potential of nanoparticles assembled by oleic acid-grafted chitosan showed good adsorption (81 ± 1.5 mV) (see Figure 2C) compared to chitosan alone and oleic acid alone. The results show a significant reduction of the potential surface of NPs after their fabrication, indicating that BSA-FAwas assembled up to surface of OA-grafted chitosan.

This result confirms the stability of this colloidal suspension for biological and environmental applications [31]. Additionally, it is a real indication for the combination of these dual structures compared to the zeta potential of both chitosan alone and oleic acid alone (see Figure 2A,B). Dynamic light scattering (DLS) investigation was also performed to gain evidence of the differences in the size of the materials used (see Figure S1 in the Supplementary Materials).

The distribution of used materials such as chitosan alone, oleic acid alone, oleic acid-grafted chitosan, and hybrid polymeric lipid protein micro/nano-particles on a scale bar were studied by DLS to describe the modification of the material size that was used during the experiment. The given result indicates that chitosan and oleic acid have high distributions before assembling and their complexes improve their uniformity. Hence, the polydispersity value (PDI) reflects the nanoparticle size distribution. In our study, PDI mostly ranged from 0.6 to 1. This wide range of values is closely related to the number of carboxyl groups of OA and the primary amino group of chitosan assembled together.

For the purpose of targeted delivery, the surface of NPs was also functionalized with FA conjugated to BSA. Their potential surface was modified after conjugation and measured at 18.6 ± 0.8 mV (see Figure 2D). This result indicates that the surface of OA-integrated chitosan was actually coated by a BSA-FA layer. Data of the BSA functionalized with FA was already published [25].

**Figure 3:**
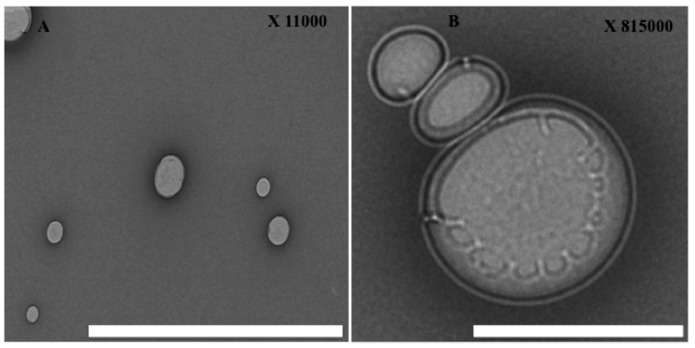
Transmission Electron Microscopy (TEM) characterization: (**A**) low magnification of HPLPNCs; and (**B**) high magnification view of HPLPNCs. Scale bars: 250 nm.

In order to characterize the structure of the hybrid nano-lipid, NPs were stained by uranyl acetate to enhance OA electron density and then imaged by Transmission Electron Microscopy (TEM). The results show a dim ring structure surrounding the core (see Figure 3) and three or four layers were completely attached having diameters of 30–60 nm [27]. These results indicated that the hydrophobic modified chitosan was well dispersed in aqueous media, with an increase in the amide linkage between chitosan and OA, and a denser hydrophobic core was formed [32]. These modifications can introduce hydrophobic groups into chitosan and form amphiphilic chitosan polymers. Some of these amphiphilic chitosan polymers can generate nano-sized self-aggregation in aqueous media [30]. In this study chitosan chains were modified by oleic acid through the introduction of the carboxylic acid group in the presence of water-soluble carbodiimide, which reacts with the carboxyl groups of fatty acids forming active ester intermediates. Consequently, the intermediates can react with primary amine groups of chitosan to form an amide bond (see scheme Figure 1).

**Figure 4:**
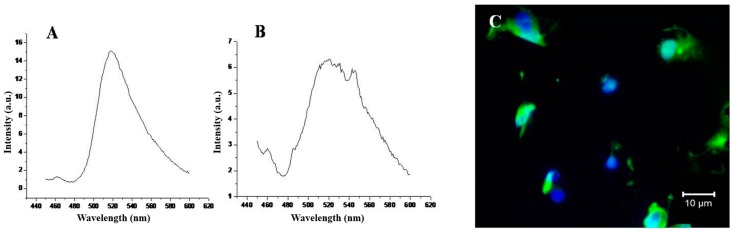
Fluorescence characterization: (**A**) Chitosan grafted oleic acid; (**B**) assembled structure of Hybrid Polymeric Lipid Protein Nano-carriers (HPLPNCs); and (**C**) cellular uptake.

**Figure 5:**
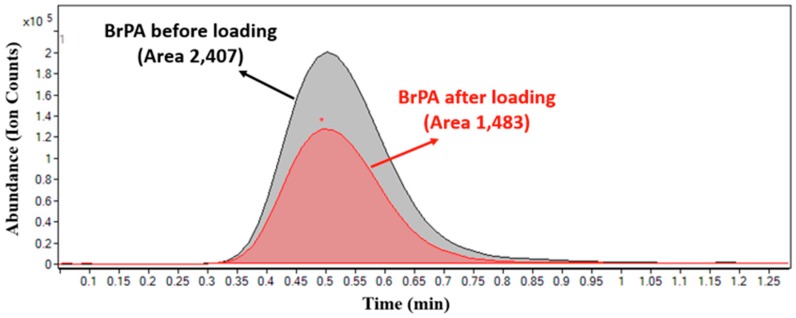
Overlapped high-resolution liquid chromatography coupled to mass spectrometry HPLC-HRMS chromatograms of BrPA before (black) and after (red) loading. The peak area was obtained from the extracted ion chromatogram (EIC) with *m*/*z* 164.9193 corresponding to the molecular ion [M − H]^−^ of BrPA. The peak obtained after loading refers to BrPA concentration in the supernatant after centrifugation.

The NP furthermore comprises three distinct functional components: (i) a hydrophobic polymeric core where chitosan was successfully entrapped inside the core and it was the main place for drug encapsulation; (ii) a hydrophilic BSA-FA shell with a delivery targeting purpose and for good liver cell binding; and (iii) a lipid monolayer at the interface of the core and the shell that acts as a molecular fence to prevent drug leakage, thereby enhancing drug encapsulation efficiency, increasing drug loading yield, and controlling drug release [33]. Moreover, the fluorescein isothiocyanate (FITC) marker integrated inside carrier moieties shows good fluorescein intensity (see Figure 4A,B). However, the intensity of FITC-labeled HPPNCs was observed by fluorescence spectroscopy showing a peak at 520 nm. Similarly, the peak emerged by FITC integrated chitosan-oleic acid moieties. Figure 4C shows that intracellular nanoparticle uptake occurred after post-cell treatment. Hence, FITC-HPPNC was visualized as a green color distributed inside the cytoplasm. Cellular uptake study using FITC-labeled HPPNCs showed that cancer cells readily accumulate the nanoparticles within cells for up to 24 h post treatment. According to Equation (1), high-resolution mass spectrometry shows good results for the encapsulation of BrPA (see Figure 5) into the NPs. Quantitative calculations provide a loading efficiency of about 0.45 mg/mL and a percentage of loading of about 45%.

### 2.2. Cellular Experiments

The cellular internalization of hybrid polymeric lipid protein carriers was measured by fluorescence microscopy. Hence, FITC-labeled carriers are successfully localized inside cytoplasm and emitted green color (see Figure 4C). The optical density measurements give an indication of the relative viable cells present at the time the dye is added.

**Figure 6:**
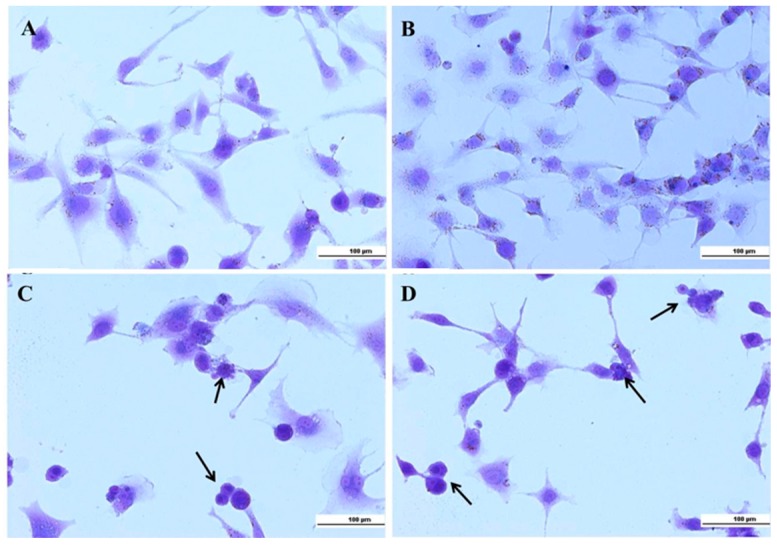
Crystal violet shows morphological characterization: (**A**) control HLF; (**B**) free HPLPNCs; (**C**) free BrPA; and (**D**) encapsulated BrPA. Arrows indicate apoptotic cells.

In this study, crystal violet was used to investigate the morphological characterization for all experimental conditions. Ethidium bromide was also used to show hyperchromatism and apoptotic bodies. Crystal violet (CV) is a triphenylmethane dye known as gentian violet, utilized widely to measure cell viability [34] or cell proliferation [35] under different conditions. Crystal violet can enter the cell membrane and reacts with cytoplasmic protein structures, distinguish between cytoskeleton and nuclear morphology. The morphological structure of either HLF cells incubated in normal conditions or that were incubated with free Hybrid Polymeric Lipid Protein Nanocarriers HPLPNCs appeared in well-organized structures with intact nuclei (see Figure 6). Most of the HLF cells incubated with free BrPA or encapsulated BrPA exhibited round and condensed structures with apoptotic morphology.

Ethidium bromide, a DNA binding dye, stains those cells that have lost their nuclear membrane integrity [36]. It is commonly used to visualize nuclear membrane disintegration and apoptotic body formations that are characteristic of apoptosis.

**Figure 7:**
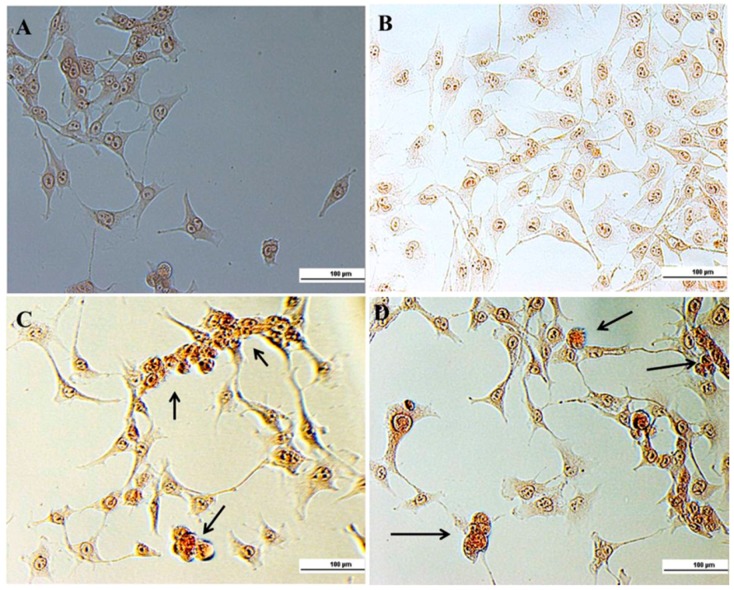
Hyperchromatism and apoptotic bodies: (**A**) control HLF; (**B**) free HPLPNCs; (**C**) free BrPA; and (**D**) encapsulated BrPA. Arrows indicate apoptotic bodies.

In Figure 7 control HLF cells and free HPLPNC showed rounded nucleus with one or more nucleoli. On the other side, hyper chromatic cells were characterized by the condensed yellow color in both free BrPA and encapsulated BrPA groups, in addition to apoptotic bodies, were seen in encapsulated BrPA slides.

**Figure 8:**
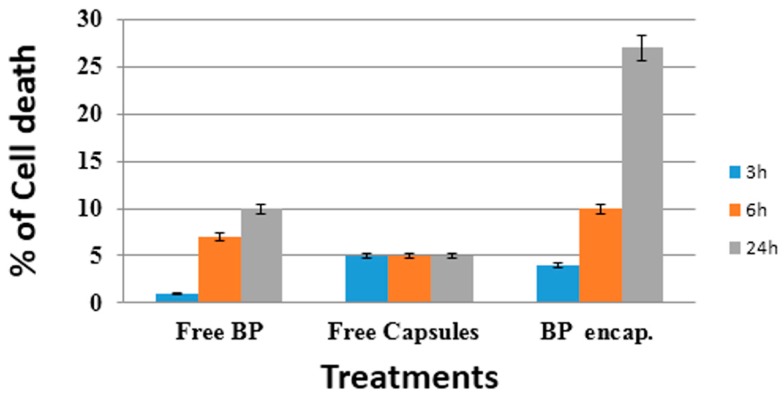
Trypan blue spectrophotometrical viability analysis against HLF cells. Percentage of dead cells upon increasing time (3–6–24 h) after treatment with free HPLPNC, free BrPA and encapsulated BrPA are reported. Data showed is an averaged value of three successive measurements with standard deviation (S.D.).

Trypan blue is one of the most commonly used methods for assessment of viability in a given cell population [37,38]. It is used in our study to quantify dead cells by spectrophotometry at 570 nm [31]. Optical analysis of the cultures revealed an admixture of live (trypan blue negative) and dead (trypan blue positive) in experimental condition. In Figure 8 the cell mortality measured by trypan blue assay was increased in case of free BrPA and also encapsulated BrPA, compared to control HLF cells and cells treated by free HPLPNC (capsules). Furthermore, upon increasing the treatment time (from 3 to 6 to 24 h) there is also a clear increase in cell death. In our previous work [25], and in this study, are reported the potential therapeutic effectiveness of targeted nanoparticles against cancer cells since they can allow smart chemotherapeutics to be accumulated in specific tumor sites, in order to minimize the potential side effects of chemotherapies on healthy cells, too. These advantages have received significant attention due to the overexpression of specific receptors, antigens, and molecules on cancer cell membrane. These molecules can be recognized by nanoparticles that were designed by folic acid [25], transferrin [39], short oligonucleotides of RNA or DNA that can fold into various conformations and engage in ligand binding [40], specific antibodies [41], and peptides [42].

## 3. Materials and Methods

### 3.1. Chemicals

Chitosan oligosaccharide (Molecular Weight (MW) 5kDa), oleic acid, and 1-ethyl-3-(3-dimethylaminopropyl) carbodiimide (EDAC), dimethyl sulfoxide (DMSO), bromopyruvate, bovine serum albumin, folic acid, trypan blue, crystal violet, and ethiduim bromide were purchased from Sigma-Aldrich (Milan, Italy).

### 3.2. Carrier Fabrication

Step 1: 1 mL of oleic acid was dissolved in 10 mL of ethanol under sonication for 15 min, then heated in water bath at 60 °C for 2 h in the presence of EDAC and fluorescence isothiocyanate. Afterwards 0.5 mg chitosan was dissolved in 50 mL of 1% acetic acid. Then 5 mL of oleic acid was mixed with 25 mL of chitosan under rotation for 15 min.

Step 2: 65 mg of folic acid was dissolved in 2.5 mL of DMSO for 30 min. Then 30 mg of EDAC and 38 mg of NHS were added, completing rotation for 1 h. Afterwards 4 mg of BSA were dissolved in 50 mL of distilled water in the presence of EDAC for 30 min. At the end 0.5 mL of activated FA mixed with 25 mL of activated BSA under rotation for 30 min.

Step 3: Chitosan integrated oleic acid was coated by BSA conjugated with FA under rotation for 30 min. Then the mixture was centrifuged at 5000 rpm for 30 min at 20 °C. Afterwards the upper layer was separated and dissolved in 10 mL Milli Q water and the mixture was dialyzed against milli Q water overnight.

### 3.3. Characterization

#### 3.3.1. Transmission Electron Microscopy (TEM)

Samples for TEM analysis were obtained by drop-casting a few microliters of solution onto standard TEM carbon-coated Cu-grids, and by allowing the solvent to fully evaporate. Samples were imaged by using a JEOL JEM 1011 TEM microscope (JEOL, Inc., Peabody, MA, USA) operating at 100 kV.

#### 3.3.2. Fluorescence Spectrophotometry

The intensity of fluorescence markers was detected by Cary Eclipse fluorescence spectrophotometer (Agilent, Santa Clara, CA, USA). The analysis was performed on the following: chitosan integrated oleic acid-FITC and free hybrid assembly.

#### 3.3.3. Zeta Potential Measurements

The zeta potential surface of carrier fabrication was measured by using a Malvern Nano ZS90 (Malvern Instruments, Malvern, UK). An average of five successful runs was considered for analysis.

#### 3.3.4. Quantification of BrPA loaded HPLPNCs by Using HPLC-Mass Spectrometry (HPLC-MS)

Briefly, BrPA solution of known concentration was incubated overnight with HPLPNCs. Then, the supernatant was removed by centrifugation and it was analyzed by high-resolution liquid chromatography coupled to mass spectrometry (HPLC-HRMS). The loading percentage was defined as the residual BrPA moles in solution after loading divided by the moles of BrPA in solution before loading. In particular the encapsulation efficiency (% loading) was calculated as the relative difference between 3-BrPA concentrations before and after the incubation experiment (see Equation (1)).

(1) % Loading = 100 × ([BrPA]i−[BrPA]f)/[BrPA]i

[BrPA]i is defined as the initial concentration of BrPA, [BrPA]f is defined as the final concentration of BrPA. HPLC-HRMS experiments were performed with an Agilent 6540 quadruple time-of-flight (QToF) mass spectrometer (Agilent, Santa Clara, CA, USA) equipped with an electrospray ionization (ESI) source and interfaced to an Agilent 1200 modular high-performance liquid chromatograph consisting of a binary pump, a vacuum degasser, a thermostated autosampler, and a thermostated column compartment. The following general conditions were adopted: ESI source operating in negative mode; solvent: 80% water (0.1% formic acid) and 20% acetonitrile; flow rate: 0.2 mL·min^−1^; drying gas (N_2_): 11 L·min^−1^; nebulizer pressure: 45 psi; drying gas temperature: 350 °C; capillary voltage: 4000 V; fragmentor: 150 V; mass range: 50–1600 *m*/*z*. Mass spectrometry chromatograms were acquired and analyzed using Agilent Mass Hunter Qualitative Analyses version B.01.04 data processing software (Agilent, Santa Clara, CA, USA). A four non-zero point calibration curve was built for BrPA by plotting the concentration of the analytical standard in aqueous solution (0.1, 0.2, 0.5, and 1.0 mg/mL) against and the peak area of the *m*/*z* 164.9193 corresponding to the extracted ion chromatogram (EIC) of BrPA. The calibration equation was: *y* = 5·106 *x* + 495,851 with a coefficient of determination *R*^2^ = 0.9979.

### 3.4. Cellular Experiments

#### 3.4.1. Cellular Studies

HLF cell lines were purchased as described in [43,44] and were maintained in DMEM medium supplemented with FBS (10%), penicillin (100 U·mL^−1^ culture medium), streptomycin (100 mg·mL^−1^ culture medium), and glutamine (5%). Cells were grown in an incubator at 37 °C, under 5% CO_2_, and at 95% relative humidity. Cell lines were serum-starved for 24 h before any test.

#### 3.4.2. Cellular Uptake

HLF Cell lines were seeded on sterilized glass coverslips into petri dishes, with a density of 2000 cells. They were grown under normal condition as previously described. After 24 h, 100 µL of hybrid assembly were added. Cellular uptake was measured after the next 24 h incubation by fluorescence microscopy.

#### 3.4.3. Crystal Violet

Ten thousand HLF cells were seeded in 24 multi-wells and grown as previously described. After 24 h, cells were added with 100 µL of free hybrid lipid nanoparticles, or with free BrPA or with encapsulated BrPA and incubated for additional 24 h. Then, DMEM was discharged and cells were washed three times with phosphate buffered saline PBS (pH 7.2). Cells were fixed for 15 min with buffered formalin (3.7%), extensively washed with PBS (pH 7.3), and finally stained with 0.01% crystal violet in PBS. After removing excess stain, cells were incubated at PBS (pH 7.3). Optical images were captured in the bright field by using a fluorescence microscope (TCS SP5; Leica, Microsystem GmbH, Mannheim, Germany) equipped with a digital camera (Leica, Microsystem GmbH, Mannheim, Germany).

#### 3.4.4. Ethidium Bromide (EB)

Cells were washed with 1× PBS buffer (pH 7.4), fixed with absolute methanol for 10 min, and washed again with 1× PBS buffer (pH 7.4). Cells were stained with 50 µL of EB (100 µg/mL) for 10–15 min and then they were immediately washed with PBS and observed under a light microscope.

#### 3.4.5. Trypan Blue

According to procedure used by Uliasz and Hewett, 2007 [12], 50 mL of sterile 0.4% trypan blue solution (final concentration 0.05%) was added to each culture well and the plate placed back into the incubator (37 °C) for 15 min. Then dye-containing media was gently removed by washing (3 × 750 mL) with ice-cold phosphate buffered saline (0.01 M PBS). A slow, steady wash prevents loss of injured cells that may originate from mechanical handling. Visual analysis of the cultures showed a mixture of live (trypan blue negative) and dead (trypan blue positive) cells in each experimental condition. Cells were then lysed with 200 mL of sodium dodecyl sulfate (SDS; 1% *w*/*v*) and the contents gently fractured taking care not to introduce air bubbles. At the end, 175 mL of the SDS: trypan blue solution was transferred to a 96-well culture dish and measured spectrophotometrically at 590 nm.

## 4. Conclusions

Chitosan-grafted OA was used as a vehicle to encapsulate BrPA through electrostatic reaction of amino–hydroxyl groups. Hence, the OA layer acts as a molecular fence to prevent drug release. Finally HPLPNCs were fabricated with smart properties, such as nano-sized diameter, spherical shape, control drug release properties, good drug capacity, and dual combination targeting. Crystal violet and ethidium bromide results confirm efficiency of encapsulated BrPA, compared to HPLPNCs alone.

## Supplementary Materials

The following are available online at http://www.mdpi.com/2079-4991/8/1/34/s1.
