# Supplementary material for: Inihibition of Glycolysis by Using a Micro/Nano-Lipid Bromopyruvic Chitosan Carrier as a Promising Tool to Improve Treatment of Hepatocellular Carcinoma
Source: Nanomaterials (Basel). 2018 Jan 10;8(1):34. doi: 10.3390/nano8010034 (PMC5791121; doi:10.3390/nano8010034)
Supplement: Supplementary file 1 [file nanomaterials-08-00034-s001.pdf]

Supplementary Material

Inihibition of Glycolysis by Using a Micro/Nano-Lipid Bromopyruvic Chitosan Carrier as a Promising Tool to Improve Treatment of Hepatocellular Carcinoma

Nemany A. Hanafy [1,2,†], Luciana Dini [3], Cinzia Citti [1,3], Giuseppe Cannazza [3,4] and Stefano Leporatti [1,*]

1 CNR NANOTEC-Istituto di Nanotecnologia, 73100 Lecce, Italy; nemany.hanafy@nanotec.cnr.it (N.A.H.); cinzia.citti@gmail.com (C.C.)
2 Department of Mathematics and Physics "E. De Giorgi", University of Salento, 73100 Lecce, Italy
3 Department of Biological and Environmental Sciences and Technologies (DiSTeBA), University of Salento, 73100 Lecce, Italy; luciana.dini@unisalento.it (L.D.); giuseppe.cannazza@unimore.it (G.C.)
4 Life Science Department, University of Modena e Reggio Emilia, 41121 Modena, Italy
† Present Address: Sohag Cancer Center, 82511 Sohag, Egypt; and Institute of Nanoscience and Nanotechnology, Kafrelsheikh University, 33516 Kafr ElSheikh, Egypt.
* Correspondence: stefano.leporatti@nanotec.cnr.it; Tel.: +39-0832-319829

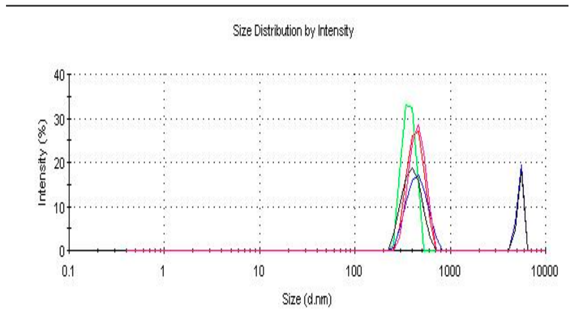

Chitosan alone

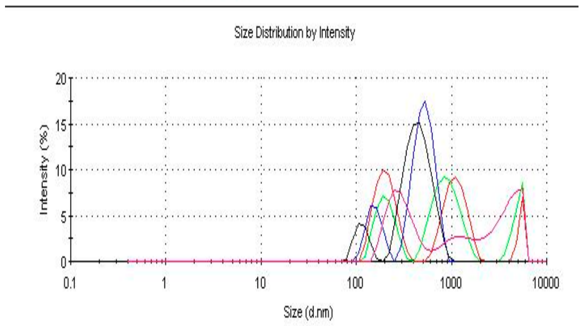

Oleic acid alone

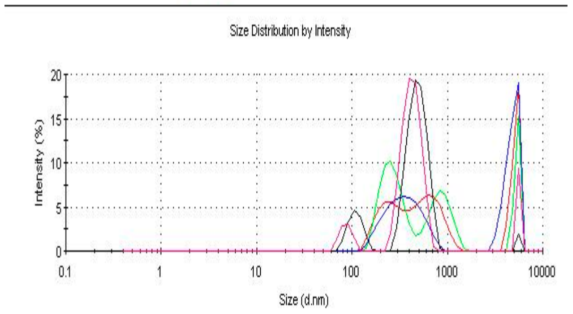

Chitosan and oleic acid NPs

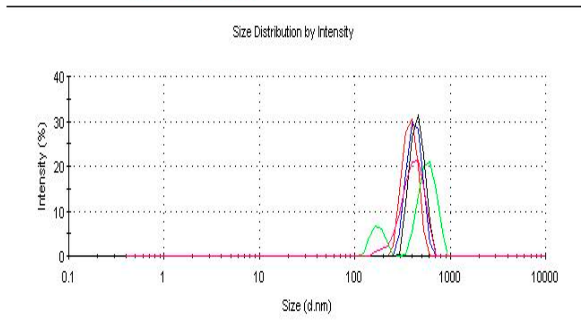

HPLPNCs

Figure S1. Size distribution of used materials. Dynamic light scattering (DLS) investigation of different materials used for producing micro/nanolipid carriers (chitosan alone, oleic acid alone, chitosan and oleic acid NPs, and HPLPNCs).
